# Canine Models for Copper Homeostasis Disorders

**DOI:** 10.3390/ijms17020196

**Published:** 2016-02-04

**Authors:** Xiaoyan Wu, Peter A. J. Leegwater, Hille Fieten

**Affiliations:** Department of Clinical Sciences of Companion animals, Faculty of Veterinary Medicine, Utrecht University, Yalelaan 108, 3584 CM Utrecht, The Netherlands; x.wu@uu.nl (X.W.); P.A.J.Leegwater@uu.nl (P.A.J.L.)

**Keywords:** copper toxicosis, nutrition, genetics, Wilson disease, Menkes disease, ATP7A, ATP7B, COMMD1, Bedlington terrier, Labrador retriever

## Abstract

Copper is an essential trace nutrient metal involved in a multitude of cellular processes. Hereditary defects in copper metabolism result in disorders with a severe clinical course such as Wilson disease and Menkes disease. In Wilson disease, copper accumulation leads to liver cirrhosis and neurological impairments. A lack in genotype-phenotype correlation in Wilson disease points toward the influence of environmental factors or modifying genes. In a number of Non-Wilsonian forms of copper metabolism, the underlying genetic defects remain elusive. Several pure bred dog populations are affected with copper-associated hepatitis showing similarities to human copper metabolism disorders. Gene-mapping studies in these populations offer the opportunity to discover new genes involved in copper metabolism. Furthermore, due to the relatively large body size and long life-span of dogs they are excellent models for development of new treatment strategies. One example is the recent use of canine organoids for disease modeling and gene therapy of copper storage disease. This review addresses the opportunities offered by canine genetics for discovery of genes involved in copper metabolism disorders. Further, possibilities for the use of dogs in development of new treatment modalities for copper storage disorders, including gene repair in patient-derived hepatic organoids, are highlighted.

## 1. Introduction

The essential micronutrient copper plays a key role in several vital biological processes including neurotransmitter synthesis, antioxidant defense, mitochondrial respiration, iron metabolism, pigmentation, and connective tissue formation [1,2,3,4,5,6,7]. As a transition metal, copper can be highly reactive, therefore copper levels need to be strictly regulated. A disruption in the function of proteins involved in the regulation of copper metabolism can lead to severe clinical phenotypes illustrated by the diseases caused by mutations in genes encoding the P-type ATPase copper transporters ATP7A and ATP7B.

Mutations in ATP7A give rise to copper deficiency disorders [8], of which Menkes disease (MD) is best described [9,10]. Menkes disease patients suffer from severe neurological impairment and failure to thrive. Treatment consists of parenteral copper supplementation, however, the disease is usually lethal in early childhood [9].

Mutations in the copper transporter ATP7B result in the copper overload disorder Wilson disease (WD) [11,12]. Wilson disease patients present with hepatitis resulting from hepatic copper accumulation and/or neurological or psychiatric symptoms [13]. Many different mutations in ATP7B may result in Wilson disease and there is a lack of a clear genotype-phenotype association [14]. Probably environmental factors or, yet unidentified, modifier genes contribute to the diverse manifestations of WD. Treatment consists of life long copper chelation therapy or liver transplantation [15,16].

Besides Wilson disease, non-Wilsonian forms or copper toxicosis leading to liver cirrhosis at young age include Indian childhood cirrhosis [17], endemic Tyrolean infantile cirrhosis [18], and idiopathic copper toxicosis [19]. In addition to genetic predisposition, increased uptake of copper via diet and drinking water predispose for development of disease. The underlying genetic defects have not been elucidated yet.

Naturally occurring copper-associated hepatitis occurs in high frequency in a number of pure bred dog populations [20]. Copper storage diseases in dogs mimic Wilson disease and ecogenetic forms of copper toxicosis with regard to hepatic copper accumulation resulting in liver cirrhosis.

One of the best described diseases is autosomal recessive copper toxicosis in the Bedlington terrier which is caused by a mutation in *COMMD1* [21]. More recently, both *ATP7A* and *ATP7B* were identified to be associated with copper toxicosis in Labrador retrievers [22]. Many more dog breeds are affected with copper toxicosis, offering the opportunity for genetic studies for identification of new genes involved in copper metabolism.

Pure bred dogs have a unique genetic structure, with large linkage disequilibrium blocks which makes them ideal for gene mapping studies [23,24]. In contrast, gene mapping studies in humans with Wilson disease or non-Wilsonian forms of copper toxicosis are difficult to perform due to low frequency of the diseases and phenotypic variability of patients. As the proteins involved in copper metabolism are highly conserved, newly identified genes in canines with copper toxicosis may be valuable for evaluation in human patients.

For the continuous development of new treatment strategies for people with copper metabolism disorders, animal models are needed. Several rodent models including naturally occurring models [25,26] as well as genetically engineered models [27,28] have proven to be of great value in the past years. Rodent models, however, have obvious limitations including short lifespan and an inappropriate body size for true longitudinal studies. As opposed to canines, a time course of liver biopsies cannot be collected from individual rodents, as they need to be sacrificed in order to obtain liver samples. Their lifespan of at most two years hampers evaluation of treatment side-effects on the long term. Canine models of copper toxicosis can be a valuable addition for development of new treatment strategies. The body size of dogs is more in the range of that of humans and facilitates translation of procedures (including those needed for application of stem-cell treatments such as catheterization of the vena porta for intraportal delivery of cell-transplants to the liver) and collection of multiple liver biopsies over time from the same animal. Furthermore, their long lifespan (up to 16 years) facilitates the evaluation of long-term treatment effects. To explore their full potential, a thorough genetic characterization of the canine models is a prerequisite, as it is in rodents.

In this review, we will discuss the present canine models of copper metabolism, and the role of the canine models in identification of new genes, the opportunities for development of new medical and dietary treatments, and the possibility of auto-transplantation of gene-corrected organoids in dogs as a large animal model for copper storage diseases.

## 2. Copper Homeostasis

**Figure 1:**
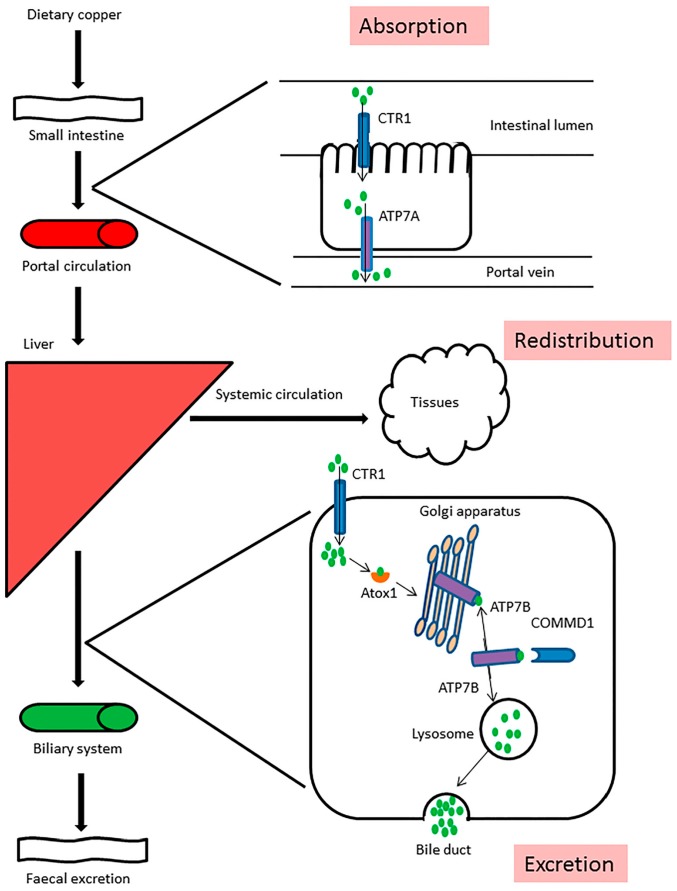
Dietary copper is absorbed in the small intestine via Ctr1. ATP7A facilitates copper transport from the enterocyte into the portal circulation for transportation to the liver. In the liver, copper is imported in the enterocytes by Ctr1. Here, copper is stored or redistributed via the systemic circulation for use in body tissues. Excretion of excess copper from hepatocytes takes place via copper transporter ATP7B. ATP7B resides in the trans-Golgi network under low copper conditions. It receives copper molecules from the chaperone ATOX1 and under high copper conditions it moves to a late endosome/lysosome compartment, from which copper is eventually excreted in the bile and expelled from the body with the feces. COMMD1 is believed to interact with ATP7B and to facilitate retrograde trafficking of ATP7B back to the trans-Golgi network.

Copper is an essential trace mineral which must be ingested from dietary sources and drinking water. Dietary copper absorption takes place in the stomach and small intestine [29]. After being released from intestinal cells, copper is transported to the liver via the portal circulation. Copper from the liver is redistributed to extra-hepatic tissues bound to ceruloplasmin, albumin, and transcuprein [30]. A minimal amount of copper is excreted via the kidney, but the main route of removal of excessive copper is via biliary excretion into the feces (Figure 1) [31]. By regulating copper storage, redistribution, and excretion, the liver is one of the main organs in homeostatic control of copper metabolism (Figure 1) [31]. Cytosolic free copper is toxic, because unbound copper generates highly reactive hydroxyl radicals that cause cell damage and lead to inflammatory reactions. Therefore, copper metabolism at the cellular level is tightly regulated to prevent the presence of free copper ions. Import of copper takes place via the plasma membrane transport protein Copper Transporter 1 (CTR1) [32]. Intracellularly, copper is bound to small copper scavengers like metallothionein (MT) [33] and glutathione (GSH) [34]. Specialized proteins, the copper chaperones, facilitate delivery of copper to various destination proteins [35]. Copper chaperone for SOD (CCS) is the copper chaperone for the detoxifying enzyme copper, zinc dependent superoxide dismutase (SOD1) [35]. SOD1 protects cells from reactive oxygen species and resides both in the cytoplasm and the intermembrane space of mitochondria [36]. Cox17 is the copper chaperone for cytochrome C oxidase (CCO), which is located in the mitochondrial inner membrane and is involved in cellular respiration [37]. Atox1 chaperones copper to the copper-transporting ATPases, ATP7A or ATP7B (Figure 1) [38]. Both ATP7A and ATP7B reside in the trans-Golgi network (TGN) and their basic functions include copper transport to newly synthesized cuproenzymes through the secretory pathway and copper export from cells [39]. In low copper circumstances, ATP7A and ATP7B reside in the trans-Golgi network, ATP7A is ubiquitously expressed in a number of cell-types, whereas ATP7B expression is detected in a selected number of cell-types, including hepatocytes. In enterocytes, copper uptake from the intestinal lumen is mediated by CTR1, which plays an important role in copper uptake by facilitating copper transport over the basolateral membrane towards the portal circulation (Figure 1). Recently, ATP7A was identified to be an important factor in mobilizing hepatic copper in response to peripheral tissue copper demand as well [40]. 

The main functions of the copper transporter ATP7B are incorporation of copper into the ferroxidase ceruloplasmin [41] and biliary excretion of excess copper [42].

In order to fulfill these functions, ATP7A and ATP7B exhibit copper responsive intracellular trafficking. Upon a rise of intracellular copper, both proteins move away from the TGN. ATP7A is mainly delivered to the cell-surface or to a post-Golgi vesicular compartment in polarized intestinal cells [43]. In hepatocytes, ATP7B is moved to an endosome/lysosome compartment in response to copper, and subsequently removes excess copper into the bile through lysosomal exocytosis towards the apical membrane of hepatocytes [44].

COMMD1, the protein that is absent in Bedlington terriers with copper toxicosis, interacts with ATP7A and ATP7B and affects trafficking of both proteins [45,46]. The role of COMMD1 in the development of copper toxicosis is not completely elucidated but evidence exists that it is associated with the regulation of the retrograde transport of ATP7B from peripheral endosomes back to the TGN in low copper circumstances (Figure 1) [47,48].

## 3. Copper Metabolism Disorders in Humans

As illustrated in the previous paragraph, copper metabolism is a complex process involving many proteins. A disruption of this tightly controlled system will lead to severe clinical phenotypes in patients. Two well documented copper metabolism disorders in humans are Menkes disease and Wilson disease which are induced by mutations of genes coding for the copper transport proteins, ATP7A and ATP7B, respectively.

MD is an X-linked recessive and lethal neurodegenerative disorder resulting from a wide spectrum of mutations in *ATP7A* [8]. Clinical symptoms result from low copper levels in the liver and brain [8] and affected MD infants suffer from brittle hair, growth failure, neurodegeneration, arterial tortuosity, and hypopigmentation. The diagnosis can be confirmed by measurement of plasma copper and neurochemical levels [49]. Treatment currently available for MD is parenteral administration of copper, which may increase lifespan, however many patients die in early childhood [50].

In contrast to MD, WD is a result of copper overload [12]. WD is a rare, autosomal recessive disease. The onset of this disease occurs at a wide range of ages from childhood [51] to the elderly [52]. Patients can present with a variety of disease phenotypes including progressive hepatic disease, neurological diseases, and psychiatric illness [53]. Diagnosis of WD is based on the combination of clinical symptoms, biochemical features, histological findings, and mutation analysis of the *ATP7B* gene. Diagnostic criteria for WD include recognition of corneal Kayser-Fleischer rings, a decreased serum ceruloplasmin level, increased excretion of urinary copper and increased hepatic copper levels (>250 mg/kg) [54]. Further, mutation analysis of the *ATP7B* gene is usually performed and over 500 mutations in the *ATP7B* gene associated with WD have currently been identified [55] and many patients are compound heterozygotes. A clear discordance between genotype and phenotypic presentation exists for WD [14]. This is illustrated by the fact that even monozygotic twins may be discordant for the WD phenotype [56]. Environmental, epigenetic, and genetic modulations probably play a role in the phenotypic expression of WD [14]. Several attempts to identify modifier genes have been made [57,58,59,60,61,62], however overall contribution of these genes to the disease heterogeneity is uncertain.

Currently, the available treatments for WD are aimed at creating a negative copper balance by copper chelation therapy or blocking of copper uptake [63]. Drugs used for copper chelation include d-Penicillamine [64] and trientine [65,66] to promote urinary copper excretion. Side effects may occur after d-Penicillamine administration including hypersensitivity, nausea, proteinuria, and development of auto-immune disorders [15]. Also, worsening of neurological signs may occur after treatment with d-Penicillamine [67,68]. Zinc salts can be used as a maintenance therapy or in asymptomatic family members of affected individuals [54]. Zinc salts can be used to decrease the absorption of dietary copper [69]. In general, Wilson disease patients are instructed to avoid foods high in copper such as chocolate, liver, nuts, mushrooms, and shellfish. However, clinical effects of an adjusted diet in the long term have not been studied. Patients are not always responsive to medical therapy, or may even show worsening of clinical symptoms [15,67]. Liver transplantation is indicated for these patients and for patients that suffer from severe liver failure due to copper-induced cirrhosis [70,71,72,73,74]. Although good outcomes in most transplantation cases have been reported [71,72,73,74], the shortage of donors, complications associated with transplantations (*i.e.*, peri-operative complications) and the risk of graft rejection, requiring long term immunosuppressive therapy, obviously complicate liver transplantation as a general cure for Wilson disease.

Non-Wilsonian disorders of copper toxicosis, often occurring in early childhood, are Indian childhood cirrhosis [17], endemic Tyrolean infantile cirrhosis [18], and Idiopathic copper toxicosis [19]. These disorders are characterized by severe hepatic copper overload and a quick progression to liver cirrhosis. Neurological phenotypes, as observed in WD, are not recognized in these infant patients. The inherited origin of these diseases was indicated by pedigree studies [18]. However, the causal genes contributing to the non-Wilson copper overload diseases have not been identified yet. Increased dietary copper uptake was assumed to be associated with development of disease, although this subject was recently under debate [75].

## 4. Copper Metabolism Disorders in Dogs

In addition to the copper disorders in humans, hereditary copper metabolism disorders are recognized in other mammals including rodents [76], sheep [77], and dogs [20]. Copper toxicosis in a variety of dog breeds mimics copper overload disorders in humans. Like in humans, copper in dogs is absorbed from diet and drinking water and subsequently accumulates in the liver. In dogs, the diagnosis is made by histological evaluation and copper measurement in liver biopsies. Hepatic copper concentrations in dogs are normally higher than in humans, with a concentration of <400 mg/kg dry weight liver (dwl) being considered as normal [78]. Copper accumulation in affected dogs can range from 800 to 10,000 mg/kg dwl. Hepatic copper accumulation induces cell death, bridging fibrosis and will progress to liver cirrhosis and hepatic failure. The disease can also manifest more acutely as fulminant liver failure. In Bedlington terriers, massive release of copper into the circulation may lead to hemolysis and anemia. In other breeds, usually a more gradual hepatic copper accumulation occurs initially without overt clinical signs. Usually during middle age (median age 7 years, range 2–12 years) dogs will display clinical symptoms of liver failure including icterus, ascites, anorexia, vomiting, and development of hepato-encephalopathy. Besides neurological signs related to hepato-encephalopathy, obvious neurological signs, or behavioral changes were not recognized. Like in humans, copper toxicosis in dogs can be treated with copper chelators d-Penicillamine [79,80] and 2,3,2-tetramine [81], or with zinc-acetate [82].

One of the best described forms of copper-associated hepatitis in dogs is copper toxicosis in Bedlington terriers [83,84]. Bedlington terrier copper toxicosis is an autosomal recessive disease resulting from a deletion of exon 2 of *COMMD1*, encoding COMM domain-containing protein 1 [21,85]. The total lack of COMMD1 protein [86] results in massive hepatic copper accumulation, which can be more than 25 times higher than normal [87]. The *COMMD1*-deficient dog is now an established canine model for copper-induced human chronic hepatitis [88].

Labrador retrievers have been reported to suffer from copper-associated chronic hepatitis with high frequency [89,90,91]. In this breed a female predisposition and a polygenic inheritance pattern with high heritability for copper accumulating traits was observed [89,90,91,92]. Labrador retrievers do not have the exon 2 deletion in the *COMMD1* gene. Illustrating that copper-associated hepatitis has a different genetic origin in different dog breeds.

Besides a genetic predisposition to disease, a large influence of dietary intake of copper and zinc on hepatic copper levels was observed in Labrador retrievers. Although the etiology of copper toxicosis in Labrador retrievers is multifactorial, a genome wide association study showed a clear association of hepatic copper levels with variations in the Wilson disease gene *ATP7B*. Interestingly, not only the Wilson disease gene but also the Menkes disease gene was associated to variation in hepatic copper levels in this breed. The presence of the mutation in *ATP7A* seemed to attenuate hepatic copper levels in Labrador retrievers, but did not induce obvious copper deficiency symptoms in this cohort. Functional assays in cell lines showed that the *ATP7B* mutation in the conserved arginine (ATP7B:p.Arg1453Gln in canine protein, corresponding to ATP7B:p.Arg1415Gln in human protein) resulted in an aberrant retention of the protein in the endoplasmic reticulum in high copper circumstances. The *ATP7A* mutation (ATP7A:p.Thr327Ile) did not induce aberrant trafficking of the protein, yet lead to abrogation of copper efflux in dermal fibroblasts, indicating a functional impairment of the protein [22].

The elucidation of part of the genetic background of copper-associated hepatitis in the Labrador retrievers, poses this breed as a new large animal model for Wilson disease. Furthermore, based on the observations in the Labrador retrievers, mutations in *ATP7A*, with a subtle effect, may be involved as modifiers in Wilson disease and may explain part of the observed difference in *ATP7B* mutation frequency and presence of clinical disease [22].

Copper storage disorders are present in a number of other pure bred dog populations including Skye terriers [93], West highland white terriers [94], Dobermanns [80], and Dalmatians [95]. The clinical phenotypes present in these dog breeds are slightly different compared to Bedlington terriers and Labrador retrievers. Future gene mapping studies in these dog breeds offer the opportunity to identify new genes and to unveil more complex copper associated phenotypes.

## 5. The Power of Gene-Mapping Studies in Dog for Identification of Genes Involved in Copper Metabolism

Illustrated by the examples of the identification of *COMMD1* in the Bedlington terriers and *ATP7A* and *ATP7B* in the Labrador retrievers, gene mapping studies in pure bred dog populations affected with copper toxicosis are a powerful tool to identify genes involved in copper metabolism. Genes involved in copper metabolism are highly conserved among species, implicating that identified genes and mutations in dogs are potentially valuable for evaluation in human patients.

One of the tools that can be used in dogs is the Genome Wide Association Study (GWAS) [96]. Typically, GWAS focuses on association between phenotype of diseases and single nucleotide polymorphisms (SNPs). SNPs are one base-pair variations in the DNA with variability in the population and a known chromosomal location. Phenotypic traits can be a binary (affected *vs.* unaffected) as well as quantitative (*i.e.*, hepatic copper levels). By using statistical methods, an association between a SNP variation and a phenotypic trait can be identified. The associated SNPs are considered to mark a region of the genome which influences the risk for disease or variation in a quantitative trait. Consecutive in-depth genetic analysis of the identified region by sequencing is needed for identification of the disease-causing variations [96].

Pure bred dogs possess some preferable characteristics for gene mapping studies [23,24,97,98]. Due to severe selection on external characteristics pure bred dogs have a simple genome build which is advantageous for unveiling the molecular genetics of complex diseases [97]. The linkage disequilibrium in dogs can extend over as much as 100 times longer distances than in humans, which reduces the number of SNPs that is needed for successful mapping of a phenotypic trait. Furthermore, the phenotypes of copper disease in a particular dog breed are much less diverse compared to those of human copper metabolism disorders, which makes accurate diagnosis much simpler.

The success of gene mapping studies in the Bedlington terrier (monogenetic disease) and the Labrador retriever (complex genetic disease) illustrate the power of the use of dogs in genetic studies for identification of new genes or modifier genes that may be involved in copper metabolism disorders in humans. GWAS in other breeds like Dobermanns, West Highland White terriers, and Dalmatians may help unveil other genes.

## 6. Canine Models for Development of New Chelation and Dietary Treatments

### 6.1. Chelation Therapy

Dogs with copper toxicosis are similar to humans with WD and ecogenetic forms of copper toxicosis with regard to hepatic copper accumulation [20]. Both in humans and in dogs, chelation therapy is the most important treatment for decreasing hepatic copper levels [79]. d-Penicillamine has been successfully used in Bedlington terriers [83], Dobermans [99], and Labrador retrievers [89] to reduce copper hepatic copper level and inflammatory lesions. Side effects from life-long systemic chelation therapy in humans may result in a decreased therapy compliance, and progression of Wilson disease [15]. Furthermore, current available chelation therapies may be ineffective for controlling clinical symptoms, or may even lead to deterioration of the disease [54]. In order to overcome the disadvantage of the current available chelators, the need exist to develop new chelators and test them for effectiveness and safety before application in human patients.

An example of a new promising drug is the small copper-binding peptide Methanobactin (MB) which was derived from methane-oxidizing bacteria [100].This unique protein has a very strong affinity for copper, and promotes copper excretion via the bile rather than via urine, which is the natural route for copper excretion. MB has shown its potential in a rat model for WD, where intraperitoneal infection resulted in a prompt, significant release of copper associated with MB into bile [101]. Intraperitoneal injections for long-term treatment in humans is not feasible. To test other routes of administration and to evaluate long-term effects of treatment, the canine models with longer life spans and larger body sizes can fulfill an important role in pre-clinical studies.

### 6.2. Dietary Strategy

Levels of dietary copper and zinc were identified to be of large influence on hepatic copper levels in Labrador retrievers [102].

This observation was explored further by the use of an adjusted diet in therapeutic protocols for dogs in a pre-clinical phase [103] and as a maintenance therapy in dogs successfully treated with d-Penicillamine [104,105]. Conclusions from these studies were that diet adaptation alone may be enough to normalize hepatic copper in a subpopulation of affected Labrador retrievers [103] and that dietary treatment may be a valuable alternative to lifelong, continuous d-Penicillamine therapy in this dog breed [104]. At this moment, diet trials were only performed in the Labrador retriever dog breed. Dietary effects on hepatic copper levels in other dog breeds affected with copper toxicosis require further study. Wilson disease patients are generally advised to avoid food with a high copper content, however the effects of specific dietary interventions have not yet been investigated.

Diet trials in canine patients illustrate the value of a large animal model for long-term dietary studies in which multiple liver biopsies over several years can be collected and evaluated. Such long-term trials are impossible in rodent models, due to their short lifespan. The small body size of rodents precludes the possibility for collection of follow-up liver biopsy specimens. Dogs can fulfill an important role for pre-clinical investigation of dietary components that may be beneficial for human patients. Dog food can be easily standardized and dietary intake can be controlled. In the near future, other dietary components, including soy protein isolates [106,107], can be evaluated in dogs prior to possible application in human patients.

## 7. Organoids and Transplantation Studies

Liver transplantation is indicated for Wilson disease patients refractory to chelation therapy or with fulminant liver failure [108]. The drawbacks of this procedure are its invasiveness, the limited availability of suitable liver transplants and the risk of graft rejection [109]. An alternative way for whole organ transplants is cell-based therapy, including transplantation of mature hepatocytes or adult stem-cells. Transplantation of primary hepatocytes is hampered by the lack of a sufficient supply of viable cells, due to the quick loss of viability and dedifferentiation in cell-cultures [110].

Recently, a long-term stable hepatic stem cell culture in 3D (hepatic organoids) was established [111,112]. Hepatic organoids are derived from the adult stem cell niche from the liver, the so called hepatic progenitor cells. Hepatic organoids are easily expandable and are stable in culture for several months which facilitates bulking of cells for re-transplantation [111,112]. To develop and evaluate new stem-cell based treatment strategies, relevant animal models are needed for testing the safety and efficacy of stem-cell treatment. In this way, canine models are invaluable to build the bridge between rodent models and human patients. Recently, a canine hepatic organoid culture was established for this purpose [113].

In order to avoid rejection of transplanted cells, autologous cell transplantation is preferable. Hepatic progenitor cells derived from a patient can be expanded in vitro before re-transplantation. Obviously, for patients with hereditary defects, gene-correction of cells before re-transplantation is then a necessary intermediate step.

Recently, we successfully demonstrated that gene supplementation, using lentiviral transduction, in hepatic organoids derived from *COMMD1* deficient dogs could rescue the copper accumulation phenotype [113]. Currently, the first experiments involving autologous organoid transplantations with *COMMD1* gene correction are being conducted in *COMMD1* deficient dogs with copper toxicosis. Herewith, we will test the safety and effectivity of organoid auto-transplantation, after gene correction as a new treatment modality for dogs and eventually humans with hepatic copper accumulation disorders [113]. At present, there is a continuing development in genome editing technologies, including TALENs [114] and CRISPR/Cas [115] that hold exciting promises for the future.

## 8. Conclusions

Recently established canine models for copper metabolism include the *COMMD1*-deficient dogs and Labrador retrievers with copper-associated hepatitis harboring both *ATP7A* and *ATP7B* mutations. Both canine models are invaluable for development and evaluation of new treatment strategies, including dietary treatment, chelation therapy, and autologous transplantation of hepatic organoids after gene-correction. Further, other canine populations affected with copper toxicosis may be explored by gene mapping studies for identification of new genes and mutations involved in copper metabolism.
